# The Structural Stability of the Endothelial Glycocalyx after Enzymatic Removal of Glycosaminoglycans

**DOI:** 10.1371/journal.pone.0043168

**Published:** 2012-08-14

**Authors:** Ye Zeng, Eno E. Ebong, Bingmei M. Fu, John M. Tarbell

**Affiliations:** 1 Department of Biomedical Engineering, The City College of New York, New York, New York, United States of America; 2 Department of Neuroscience, Albert Einstein College of Medicine, New York, New York, United States of America; University of Patras, Greece

## Abstract

**Rationale:**

It is widely believed that glycosaminoglycans (GAGs) and bound plasma proteins form an interconnected gel-like structure on the surface of endothelial cells (the endothelial glycocalyx layer–EGL) that is stabilized by the interaction of its components. However, the structural organization of GAGs and proteins and the contribution of individual components to the stability of the EGL are largely unknown.

**Objective:**

To evaluate the hypothesis that the interconnected gel-like glycocalyx would collapse when individual GAG components were almost completely removed by a specific enzyme.

**Methods and Results:**

Using confocal microscopy, we observed that the coverage and thickness of heparan sulfate (HS), chondroitin sulfate (CS), hyaluronic acid (HA), and adsorbed albumin were similar, and that the thicknesses of individual GAGs were spatially nonuniform. The individual GAGs were degraded by specific enzymes in a dose-dependent manner, and decreased much more in coverage than in thickness. Removal of HS or HA did not result in cleavage or collapse of any of the remaining components. Simultaneous removal of CS and HA by chondroitinase did not affect HS, but did reduce adsorbed albumin, although the effect was not large.

**Conclusion:**

All GAGs and adsorbed proteins are well inter-mixed within the structure of the EGL, but the GAG components do not interact with one another. The GAG components do provide binding sites for albumin. Our results provide a new view of the organization of the endothelial glycocalyx layer and provide the first demonstration of the interaction between individual GAG components.

## Introduction

The luminal surfaces of vascular endothelium are covered by an endothelial glycocalyx layer (EGL) which serves as a transport and adhesion barrier and plays important roles in the mechanotransduction of shear stress [1]–[4]. A cartoon that integrates all of the components of the glycocalyx was described in our previous paper [2]. The glycocalyx is comprised of a variety of macromolecules, including glycoproteins bearing acidic oligosaccharides and terminal sialic acids (SA), and proteoglycans (PGs) with glycosaminoglycan (GAG) side chains. GAGs are linear polydisperse heteropolysaccharides, characterized by distinct disaccharide unit repeats. Specific combinations of these give rise to different GAG families, including heparan sulfate (HS), chondroitin sulfate (CS) and hyaluronic acid (HA, also known as hyaluronan). In the vasculature, the most prominent GAGs on the surface of endothelial cells (ECs) are HS, accounting for more than 50% of the total GAG pool, the rest being comprised of CS and HA.

The polyanionic nature of the constituents of the EGL imparts to it a net negative charge [4]. HS and CS are largely responsible for its anionic charge and are covalently linked to PGs. PGs are synthesized in the Golgi apparatus, and are either secreted, bound to the cell surface or stored in intracellular compartments [5]. A single PG core protein can bind with multiple chains of HS and CS in EGL [6]. The proportion of HS to CS chains has been shown to be on the order of 4∶1 in normal murine mammary gland epithelial cells and was reduced to about 5∶4 in the presence of transforming growth factor-β (TGF-β), although the amount of PG core proteins remained relatively unchanged [7]. In both bovine aortic endothelial cells (BAEC) and human aortic endothelial cells (HAEC), 30 mM glucose treatment resulted in a decrease of HS, but not CS or perlecan [8]. Similarly, in glomerular ECs, 25.5 mM glucose induced a 50% reduction of HS immuno-fluorescence intensity relative to 5.5 mM glucose, but no reduction in PG core proteins [9]. All of those studies suggest that HS and CS GAGs play very important roles in the structure and function of the EGL.

HA is a non-sulphated GAG that is not covalently bound to a core protein. HA is usually much longer than protein attached GAGs [10]. Long chains of HA, attached to endothelial membrane bound receptors, such as CD44, are presumed to intertwine through the glycocalyx and provide part of the scaffold for the EGL [11]. In rat vascular endothelium, HA has been shown to be scattered over the plasma membrane and concentrated in caveolae [12].

Plasma proteins also contribute to the structural organization of the EGL, including albumin and orosomucoid [13]. Studies have demonstrated the binding of albumin to the endothelial surface using techniques of electron microscopy [14], intravital fluorescence microscopy [15] and confocal fluorescence microscopy [16]. In the presence of physiological albumin concentrations, fibrinogen binding to intact BAECs is greatly diminished [17]. Our recent in vitro investigation using cryo-EM demonstrated that the morphology of the EGL is mesh-like [16]. It has been presumed that a cross-linked mesh created by the interaction among membrane-bound PGs, plasma proteins, and soluble PGs, contribute to the stability of the EGL [6].

The glycocalyx is rapidly and continuously shed and synthesized, which facilitates endothelial adaptation to changes in the local environment [18]–[20]. In disease states such as inflammation [21], [22], atherosclerosis [22], endotoxemia [23], and septic shock [24], the EGL remains in a diminished state. Recent studies explored the EGL function by direct removal of selected components of the glycocalyx with specific enzymes. Interestingly, different specific enzyme treatments resulted in distinct functions of the EGL in both mechanotransduction [25] and permeability [26], [27]. We previously demonstrated that a 2 hr pretreatment with 15 mU/mL heparinase III, 15 mU/mL chondroitinase ABC, 15 mU/mL neuraminidase, and 1.5 U/mL hyaluronidase, respectively removed 42.6% HS, 44.2% CS, 38.4% SA, and 61.7% HA in cultured BAEC, and found that shear-induced nitric oxide (NO) production was blocked in the presence of those enzymes except for chondroitinase ABC [25]. In addition, other experiments suggest a layered EGL structure with different components modulating both solute and cell penetration into the layer. For example, treatment with 140 U/mL hyaluronidase for 1 hr in hamster cremaster muscle microvessels enhanced the penetration of 70-and 145-kDa dextrans, but did not affect the exclusion of red cells and anionic proteins, showing that significant components of the barrier remained in this tissue after hyaluronidase treatment [11], [27]. On the other hand, in the same vessels, treatment with heparinase (1 mg/mL) caused a significant increase in the penetration of red cells [28]. Recently, Gao and Lipowsky [26] reported that a 10 min perfusion with 10 U/mL chondroitinase ABC and 3000 U/mL hyaluronidase solution in the post-capillary venules of the rat mesentery, significantly increased the penetration of 70 kDa dextrans, but a perfusion with 50 U/mL heparinase III decreased it. Enzymatic treatment of the glycocalyx can also facilitate adhesion of leucocytes to the endothelium [29], [30]. Additionally, Nikmanesh et al [31] demonstrated that applying 15 mU/mL heparinase III to degrade HS on embryonic stem cells, abolished the shear stress-induced expression of several characteristic EC genes: vWF, VE-cadherin, ZO-1, eNOS, and COX-2. Overall, most previous investigations focused changes in the function of the EGL after specific enzyme treatments. However, the mechanisms underlying those changes in function, and the alterations in organization and spatial distribution of glycocalyx components induced by enzyme treatments are less well understood.

It is widely believed that the negatively charged GAGs capture circulating plasma protein and form an interconnected gel-like structure in an aqueous environment [4], [32]–[34]. In the present study, we tested whether depletion of a GAG component with a specific enzyme would affect the distribution and thickness of other GAG components as well as adsorbed proteins. The hypothesis was that the glycocalyx would collapse when a GAG component was reduced below a critical level. To test this hypothesis, we used heparinase III, hyaluronidase, and chondroitinase ABC to deplete individual GAGs, and imaged HS, CS, HA and adsorbed albumin with immuno-fluorescent confocal microscopy.

## Materials and Methods

### Cell Culture

Rat fat pad endothelial cells (RFPECs) were cloned from cells originally isolated from rat epididymal fat pad (a gift from Dr. David C. Spray, Albert Einstein College of Medicine, Bronx, NY) [35]. As previously described [16], [36], RFPECs were cultured at 37°C with 5% CO_2_ in Dulbecco’s Modified Eagle Medium (DMEM, Invitrogen, USA) supplemented with 10% fetal bovine serum (FBS, Invitrogen, USA), and 1% penicillin-streptomycin. Cells between passages 22 and 30 were then seeded onto glass bottom dishes (MatTek, USA) until confluence (2∼3 days).

**Figure 1:**
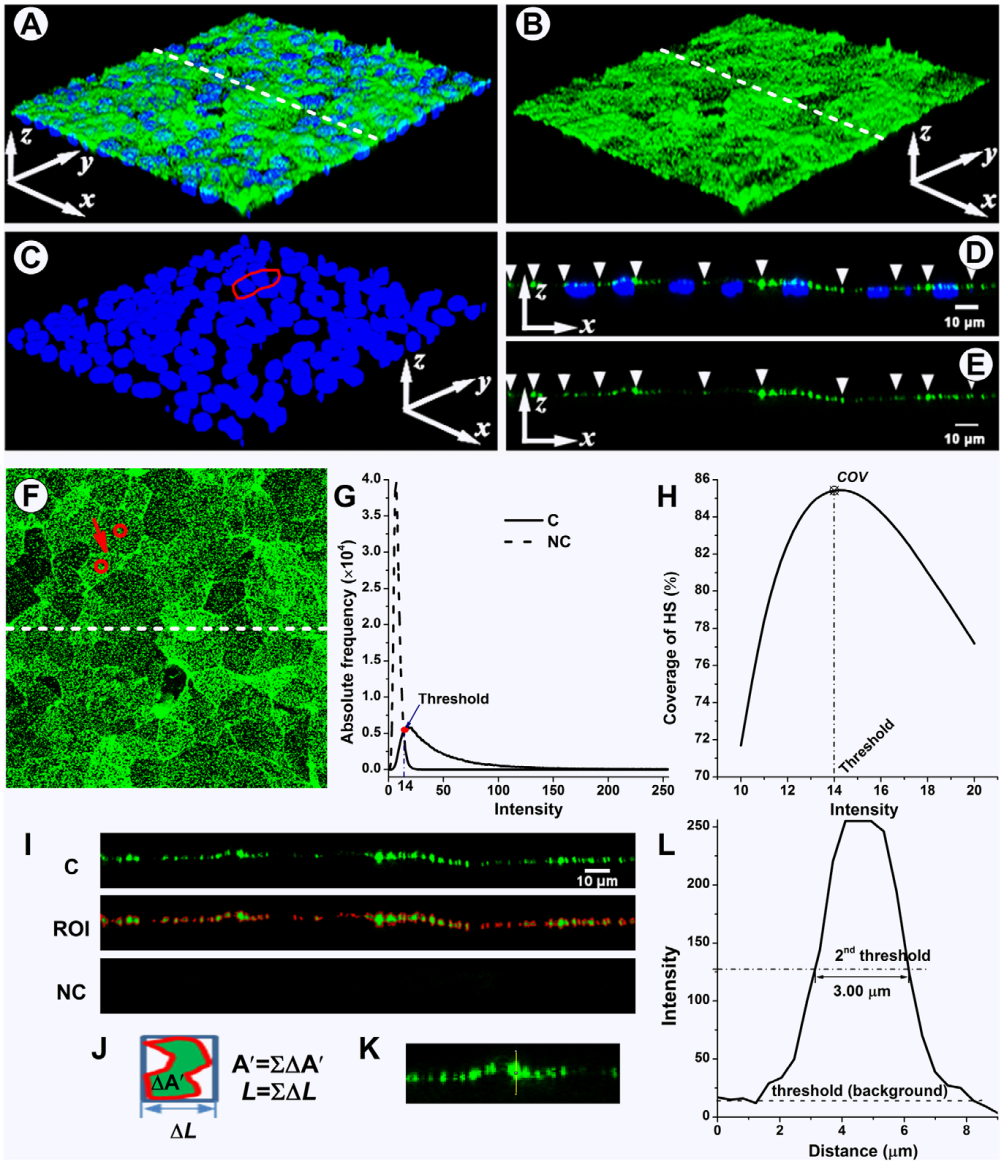
The methods for image quantification analysis. The 3D reconstruction of the Z-series stack (A), the split green channel (HS) (B), and blue channel (DAPI) (C). (D–E) XZ-slice images along the dashed line in (A) and (B). (F) The max-intensity Z-projection of HS. The red outline in (C) and the circles in (F) show the nuclei and cell body of the same two cells. The red arrow in (F) indicates the junction between these cells. The white arrow heads in (D) and (E) indicate the cell-cell junctions along the slice. (G) The frequency curves of pixel intensity from the max-intensity Z-projection images of both control and negative control (no antibody). The non-zero point of intersection between the curves was defined as the background threshold. All information equal to or higher than background was selected as a region of interest (ROI). (H) The maximum coverage was usually obtained at threshold. (I) top: the split green channel (HS) XZ-slice; middle: the red region shows the ROI in the XZ-slice at threshold; bottom: negative control, the area of the ROI in XZ-slices was usually zero at threshold. (J) The area of a region bounded by a red outline (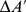) and the width of the region’s bounding box (Δ*L*). The total area and length of all regions represent the area (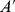) and the length (*L*), respectively, of the ROI (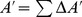;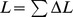). The average thickness (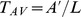) in this slice is 2.23 µm. (K–L) Partial enlargement of the XZ-slice image with a junctional thickness measure line. The half-maximum intensity value was employed as the 2^nd^ threshold for junctional thickness detection. In this image, the signal-to-noise ratio is 9.11, and the junction thickness of HS is 3.00 µm.

### Heparinase III Treatments

*F. heparinum* Heparinase III (IBEX, Canada) is selectively active only towards HS [37]. For HS staining after enzyme treatment, RFPEC monolayers were pretreated with 1 mL experimental media containing 0 (control), 15, 45, 135, 405, 1215, and 3645 mU/mL heparinase III for 2 hr. For other GAG component staining after enzyme treatment, RFPEC monolayers were pretreated with 1 mL experimental media containing 0 (control), 135, and 1215 mU/mL heparinase III for 2 hr. The experimental media was prepared using phenol red free DMEM containing 5% FBS and 0.5% bovine serum albumin (BSA, sigma), pH 7.3.

**Figure 2:**
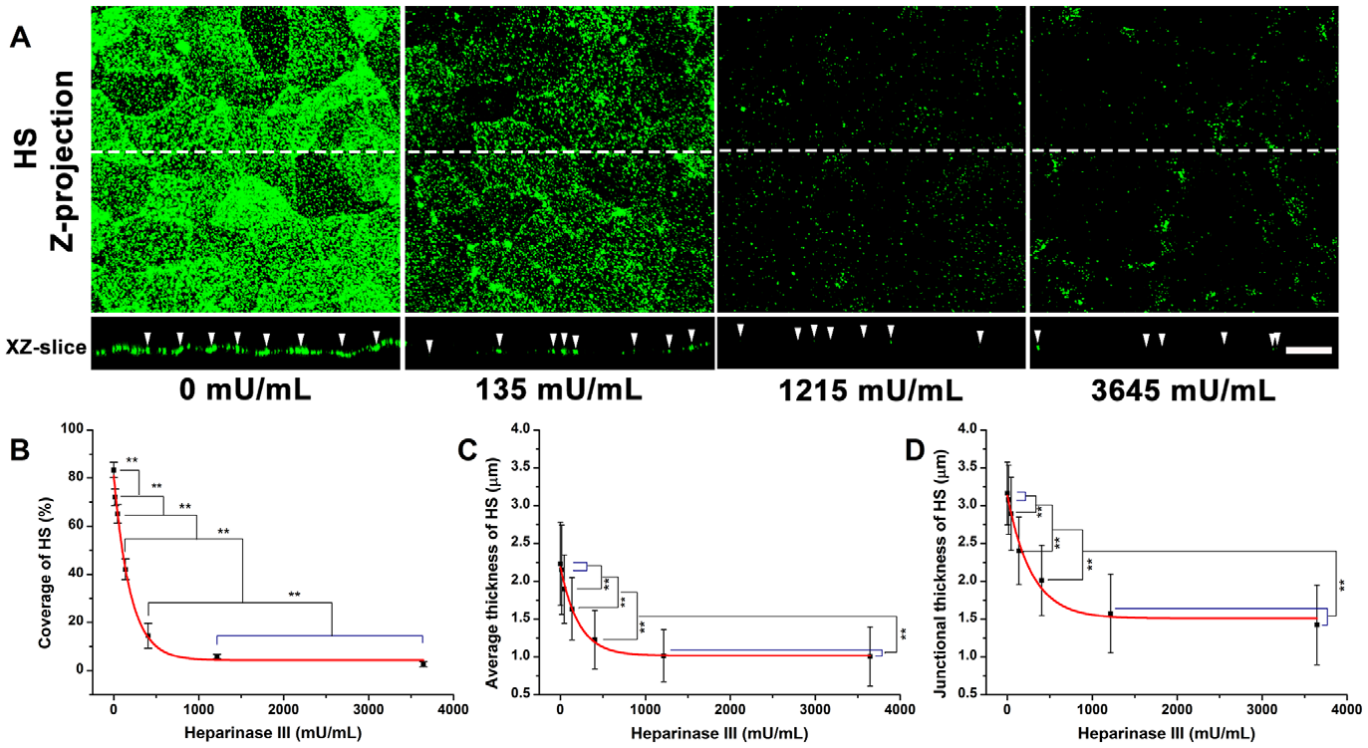
The distribution of HS on RFPECs after 2 hr heparinase III treatment. (A) top: Z-projection; bottom: cross-sectional images of stack along the dashed line. The arrow head indicates the cell-cell junction. Scale bar: 20 µm. (B) The changes in coverage; (C) the average thickness; (D) the junction thickness. HepSS-1 epitope HS-antibodies were used. The coverage, average thickness, and junction thickness were significantly decreased with increasing concentration of heparinase III. **P<0.01.

### Hyaluronidase Treatments

Although many hyaluronidases are capable of degrading CS [38], hyaluronidase from *Streptomyces hyalurolyticus* (Sigma, USA) is specific for HA and is inactive against CS. For HA staining after enzyme treatment, RFPEC monolayers were pretreated with 1 mL experimental media containing 0 (control), 1.5, 4.5, and 13.5 U/mL hyaluronidase for 2 hr. For other GAG component staining after enzyme treatment, RFPEC monolayers were pretreated with 1 mL experimental media containing 0 (control), 1.5, and 4.5 U/mL hyaluronidase for 2 hr. The experimental media was prepared using phenol red free DMEM containing 5% FBS and 0.5% BSA, pH 7.3.

**Figure 3:**
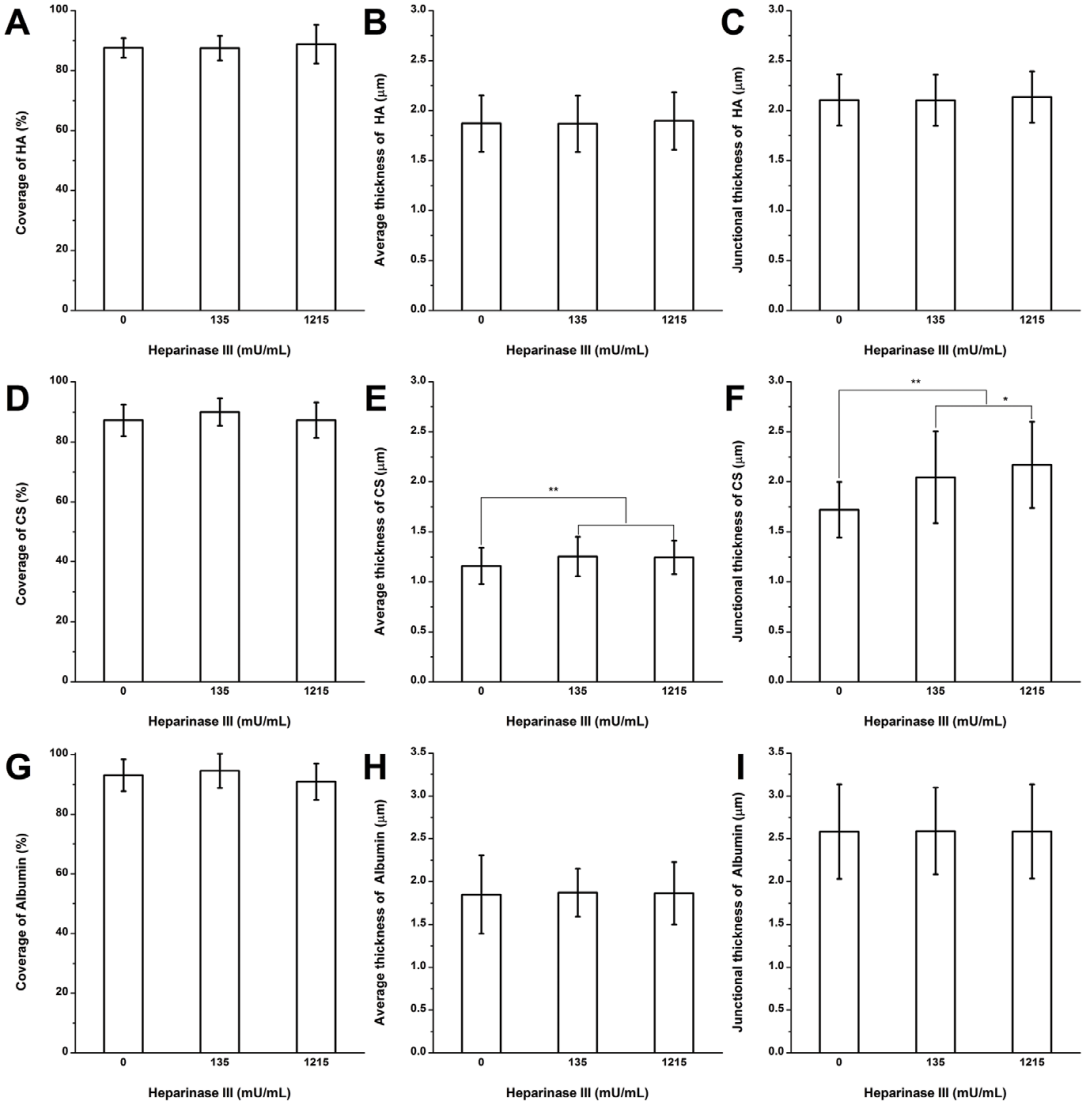
The quantification of HA, CS, and albumin on RFPECs after 2 hr heparinase III treatment. (A)–(C) The changes in the coverage, the average thickness, and the junction thickness of HA; (D)–(F) CS; (G)–(I) adsorbed albumin. *P<0.05; **P<0.01.

### Chondroitinase ABC Treatments

*P. vulgaris* chondroitinase ABC (Sigma, USA) catalyzes the eliminative degradation of CS and HA [39], [40]. Importantly, there is no chondroitinase available that reacts with CS, but not with HA. For CS staining after enzyme treatment, RFPEC monolayers were pretreated with 1 mL experimental media containing 0 (control), 15, 45, 135, and 405 mU/mL chondroitinase ABC for 2 hr. For other GAG component staining after enzyme treatment, RFPEC monolayers were pretreated with 1 mL experimental media containing 0 (control), 15, and 405 mU/mL chondroitinase ABC for 2 hr. The experimental media was prepared using phenol red free DMEM containing 5% FBS, 0.5% BSA, pH 7.3, and 60 mM sodium acetate.

**Figure 4:**
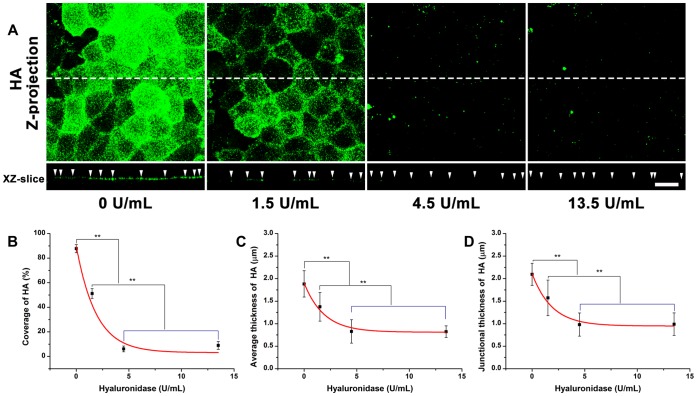
The distribution of HA on RFPECs after 2 hr hyaluronidase treatment. (A) top: Z-projection; bottom: cross-sectional images of stack along the dashed line. The arrow head indicates the cell-cell junction. Scale bar: 20 µm. (B) The changes in coverage; (C) the average thickness; (D) the junction thickness. **P<0.01.

### Immunofluorescence Staining

In order to assess structural changes of the EGL, immunofluorescence staining was performed on the enzyme treated RFPEC monolayers.

**Figure 5:**
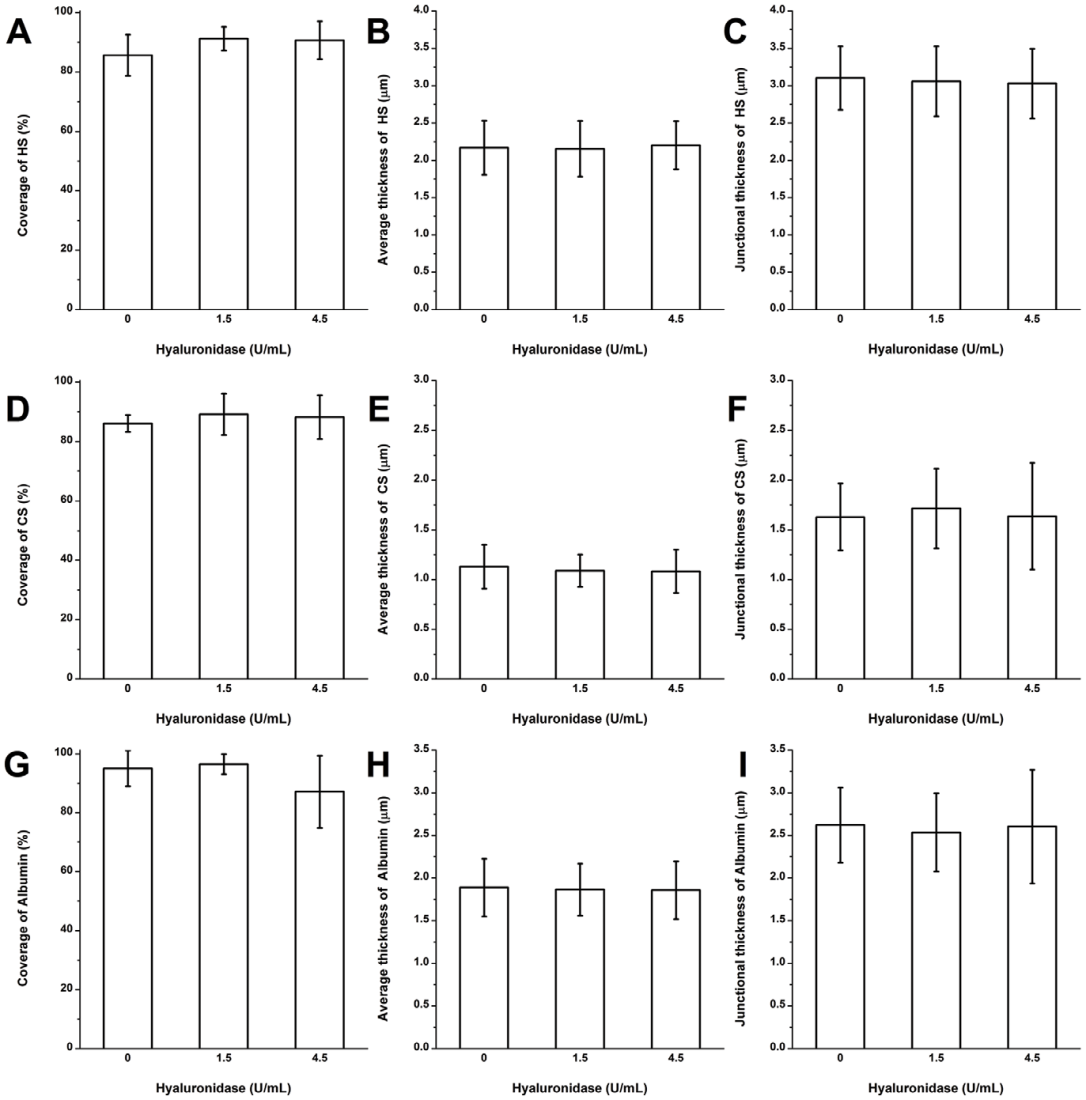
The quantification of HS, CS, and albumin on RFPECs after 2 hr hyaluronidase treatment. (A)–(C) The changes in the coverage, the average thickness, and the junction thickness of HS; (D)–(F) CS; (G)–(I) adsorbed albumin.

#### Staining of HS

The RFPEC monolayers were quickly washed one time with 1×Dulbecco’s phosphate-buffered saline (DPBS) and fixed with 2% paraformaldehyde/0.1% glutaraldehyde for 30 min at RT, then blocked with 2% Goat serum (GS, Invitrogen, USA) for 30 min followed by an overnight incubation at 4°C with mouse monoclonal primary antibody (1∶100; 10E4 epitope, AMS Biotechnology, USA; or HepSS-1, US Biological, USA), washed three times in 1×DPBS, and finally incubated with Alexa Fluor 488 goat anti-mouse secondary antibody (1∶400; Molecular Probes, USA) for 30 min at RT followed by three washes in 1×DPBS.

**Figure 6:**
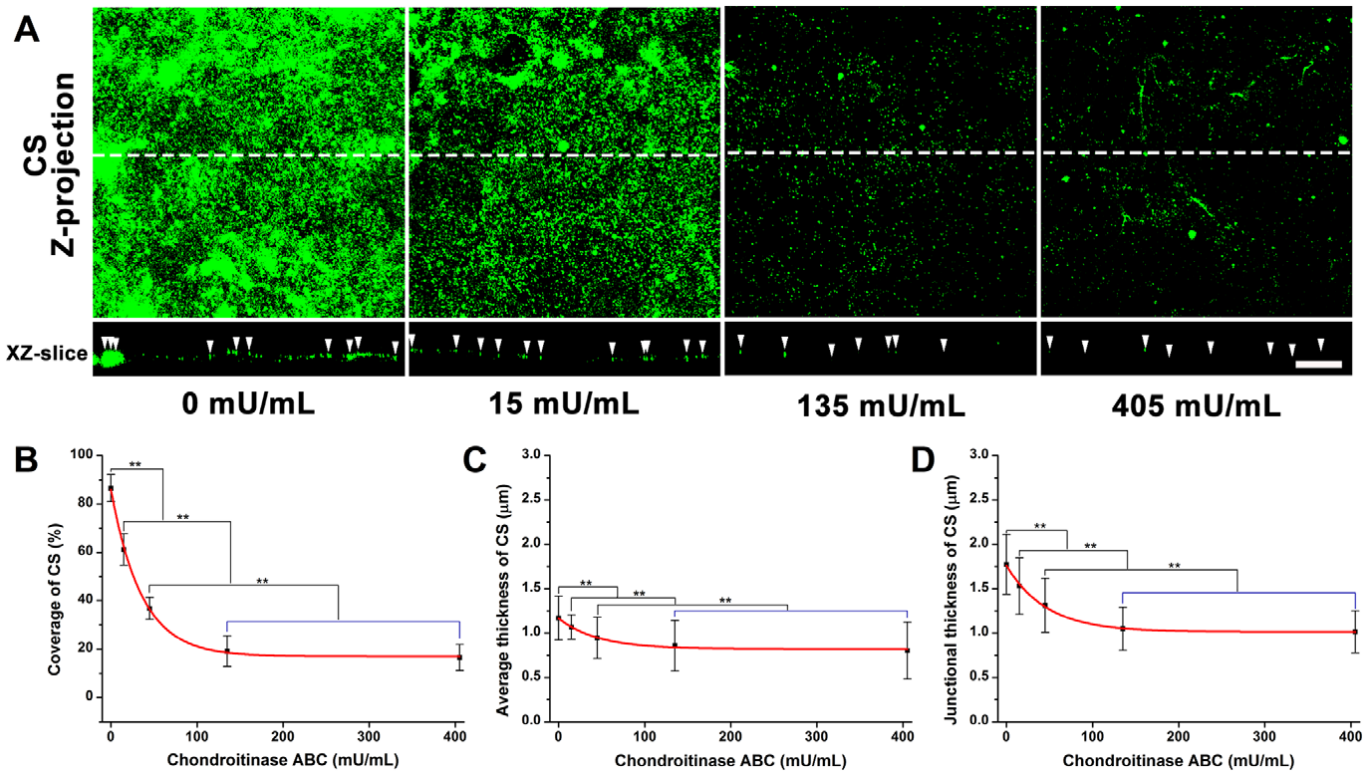
The distribution of CS on RFPECs after 2 hr chondroitinase ABC treatment. (A) top: Z-projection; bottom: cross-sectional images of stack along the dashed line. The arrow head indicates the cell-cell junction. Scale bar: 20 µm. (B) The changes in coverage; (C) the average thickness; (D) the junction thickness. **P<0.01.

#### Staining of HA

The RFPEC monolayers were quickly washed one time with 1×DPBS and fixed with 2% paraformaldehyde/0.1% glutaraldehyde for 30 min at RT, then blocked with 2% GS in 1×DPBS without calcium and magnesium for 30 min followed by an overnight incubation at 4°C with biotinylated hyaluronic acid binding protein (HABP; 50 µg/mL; EMD Chemicals, USA), washed three times in 1×DPBS, and finally incubated with Alexa Fluor 488 anti-Biotin (1∶100; Jackson ImmunoResearch Lab, USA) for 30 min at RT followed by three washes in 1×DPBS.

**Figure 7:**
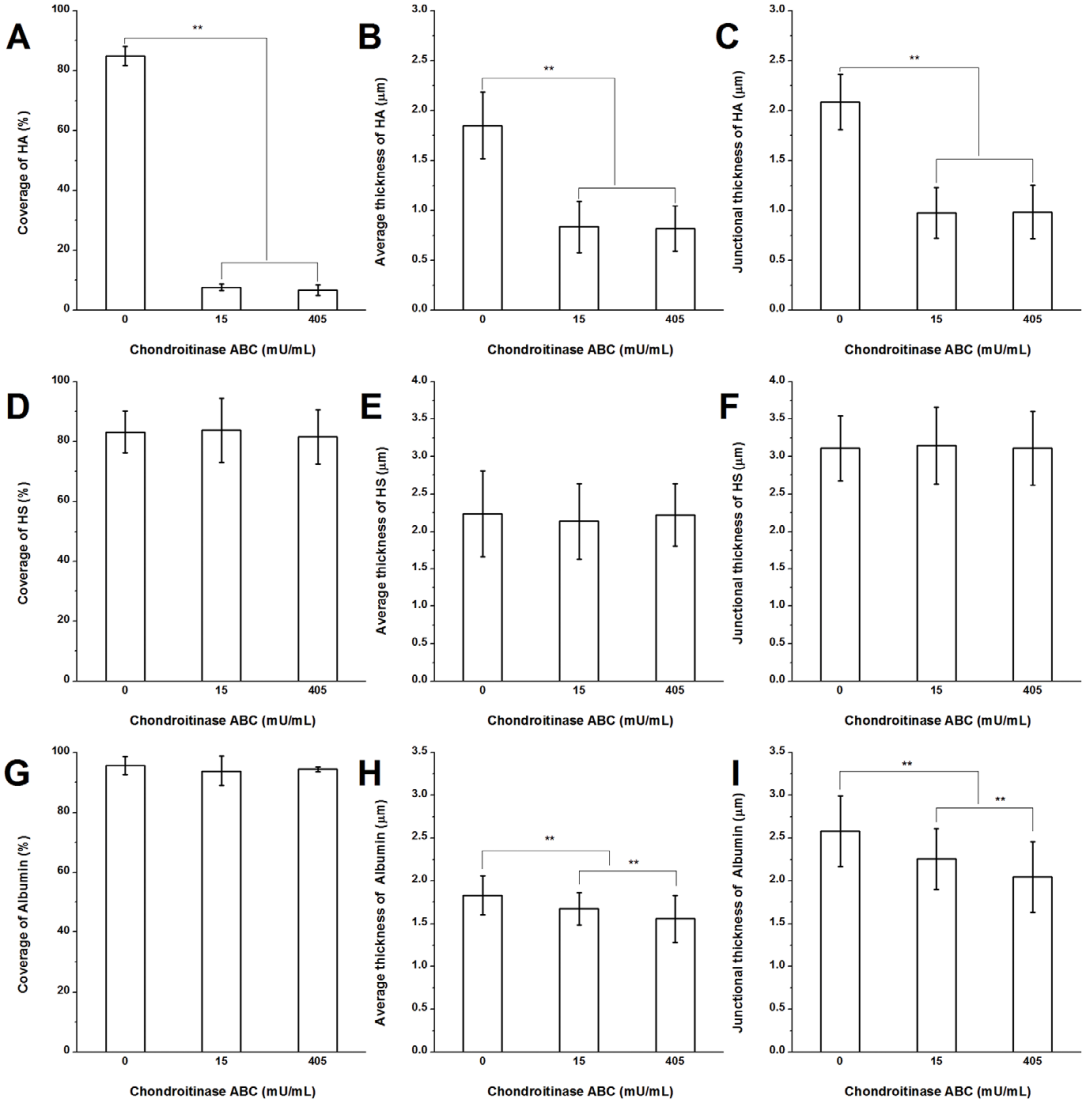
The quantification of HA, HS, and albumin on RFPECs after 2 hr chondroitinase ABC treatment. (A)–(C) The changes in the coverage, the average thickness, and the junction thickness of HA; (D)–(F) HS; (G)–(I) adsorbed albumin. **P<0.01.

#### Lectin labeling

The *B.simplicifolia* lectin II (GSL II) can recognize exclusively α- or β-linked N-acetylglucosamine residues present on CS. The RFPEC monolayers were quickly washed one time with 1×DPBS and incubated with biotinylated GSL II (100 µg/cm^2^ in 1×DPBS without calcium and magnesium; Vector Labs, USA) for 15 min on ice, then incubated for 30 min with Alexa Fluor 488 anti-Biotin (1∶100 in 1×DPBS) followed by three washes in 1×DPBS.

#### Staining of BSA

The RFPEC monolayers were quickly washed one time with 1×DPBS and fixed with 2% paraformaldehyde/0.1% glutaraldehyde for 30 min at RT, then blocked with 2% GS for 30 min followed by an overnight incubation at 4°C with anti-BSA (rabbit IgG, 1∶300; Molecular Probes, USA), washed three times in 1×DPBS, and finally incubated with Alexa Fluor 488 goat anti-rabbit secondary antibody (1∶400; Molecular Probes, USA) for 30 min at RT followed by three washes in 1×DPBS.

**Table 1:** A summary of the structural changes of the EGL after enzyme treatment.

Enzyme^a^		Tissue/Cell	Visualization method			Structural changes of EGL (I, T)^c^		Perfusion/Incubation	Ref.
	Concentration		Microscopy	Probe/Dye^b^	Target	Not treated	Treated		
**Heparinase**									
Heparinase, Sigma	1.0 U/mL in PBS	Coronary artery of rabbit	Fluorescent microscope	LRB-SC- WGA	HS,HA, SA		I:−15±2%	90 min	[53] In vitro
		Upstream of thoracic aorta					I:−28±2%		
		Downstream of thoracic aorta					I:−11±2%		
	5 U/mL in serum-free DMEM	HUVEC	CLSM	FITC-WGA	HS,HA, SA		I:−21±8%	60 min	[54] In vitro
Heparinase III, Sigma	50 U/mL in PBS	Post-capillary venules of the rat mesentery	Intravital microscope	FITC–D×70 exclusion	EGL	T:463.1±146.1 nm	T:234.0±106.0 nm (−43.3%)	10 min	[26] In situ
	15 mU/mL	BAEC	Cryo-Electron microscopy	Uranyl acetate-lead citrate	EGL	T:11.35±0.21 µm	T:11.98±0.73 µm	2 hr	[16] In vitro
	15 mU/mL in DMEM with 1%BSA		Fluorescent microscope	Anti-HS and second antibody	HS		I:−42.6±7.6%		[25] In vitro
**Hyaluronidase**									
Bovine testes, Type VI-S, Sigma	25 IU/mL	Rat myocardial capillaries	Electron microscopy	Alcian blue	EGL	T:182–512 nm	T:77–201 nm (−57.7%)	8 mL/min 1 hr	[55] In situ
	3000 U/mL in PBS	Post-capillary venules of the rat mesentery	Intravital microscope	FITC–D×70 exclusion	EGL	T:463.1±146.1 nm	T:303.3±165.8 nm (−26.1%)	10 min	[26] In situ
	100 U/mL in PBS	Carotid arteries of C57BI6/J mice	Two-photon laser scanning microscopy	FITC-WGA	HS,HA, SA	T:2.3±0.1 µm	T:Not altered	1 mL/h for 15 min	[42] In vitro
							T: 1.6 µm (−25%)	1 mL/h for 45 min	
						ALS:20.1±3.9%	Increased 3-fold	1 mL/h for 15 or 45 min	
*Streptomyces hyalurolyticus,* sigma	50 U/mL in serum-free DMEM	HUVEC	CLSM	FITC-WGA	HS,HA, SA		I:−29±3%	60 min	[54] In vitro
	1.5 U/mL in DMEM with 1%BSA	BAEC		ELISA kit from Echelon	HA		I:−61.7±4.7%	2 hr	[25] In vitro
**Chondroitinase**									
Chondroitinase ABC, Sigma	10 U/mL in PBS	Post-capillary venules of the rat mesentery	Intravital microscope	FITC–D×70 exclusion	EGL	T:463.1±146.1 nm	T:285.6±145.2 nm (−34.1%)	10 min	[26] In situ
	0.2 U/mL in PBS	Coronary artery of rabbit	Fluorescent microscope	LRB-SC-WGA	HS,HA, SA		I:−10±1%	90 min	[53] In vitro
		Upstream of thoracic aorta					I:−22±2%		
		Downstream of thoracic aorta					I:−15±2%		
	1 U/mL in serum-free DMEM	HUVEC	CLSM	FITC-WGA	HS,HA, SA		I:−13±5%	60 min	[54] In vitro
	15 mU/mL in DMEM with 1%BSA	BAEC	Fluorescent microscope	Biotinylated-B.simplicifolia lectin	CS		I:−44.2±5.6%	2 hr	[25] In vitro

IU: International unit; HUVEC: human umbilical vein endothelial cell; BAEC: bovine aortic endothelial cells; CLSM: confocal laser scanning microscopy; WGA: Wheat germ agglutinin; FITC: Fluorescein isothiocyanate; LRB-SC: Lissamine rhodamine B sulfonyl chloride;

a Unit: for heprinase III, one IU = 600 Sigma Unit; for hyaluronidase, one IU =  one National Formulary unit (Sigma used).

b Cationic probes: Alcian blue. Lectin: FITC– and LRB-SC–WGA selectively binds to N?acetylglucosamine (HS, HA) and N?acetylneuraminic acid (SA) residues [54], [56]; Biotinylated-B.simplicifolia lectin has the ability to recognize N-acetylglucosamine (CS) residues.

c The structural changes of the EGL. I: Fluorescence intensity; T: Thickness; ALS: Surface area lacking signal.

Antibodies were diluted in blocking buffer unless indicated otherwise. Finally, cells were mounted in Aqua-Poly/Mount coverslipping medium (Polysciences, USA). Negative controls were prepared without the primary antibodies or binding proteins. Detailed information about antibodies that were used in the present study is described in Discussion S1 (Figures S4, S5, and S6).

### Confocal Microscope and Data Acquisition

All samples were imaged with a Zeiss LSM 510 laser scanning confocal microscope (Confocal Microscopy Laboratory, The City College of New York, NY) using a Plan-Neofluar 40×/1.3 Oil DIC or Plan-Apochromat 63×/1.4 Oil DIC objective. The field of view (FOV) was approximately 210.4×210.4 (40×) or 133.6×133.6 µm^2^ (63×), captured into an 8 bit file with a 512×512 pixel resolution. The Alexa Fluor 488 fluorescence was excited by the 488 nm line of an argon ion laser, and filtered through the 505–550 nm band-pass emission filter. The DAPI was excited by a 405 nm diode laser, and filtered through the 420–480 nm band-pass emission filter. For each type of staining, the laser transmission and laser gain levels were adjusted just below the fluorophore saturation level, and then were applied for the acquisition of all images. The pinhole size of the Alexa Fluor 488 channel was set to 1 Airy unit for the best compromise between depth discrimination and efficiency, and then that of the DAPI channel was adjusted so that each channel had the same “optical slice". To obtain a Z-series stack, the interval between slices was either 0.3 or 0.36 µm.

**Figure 8:**
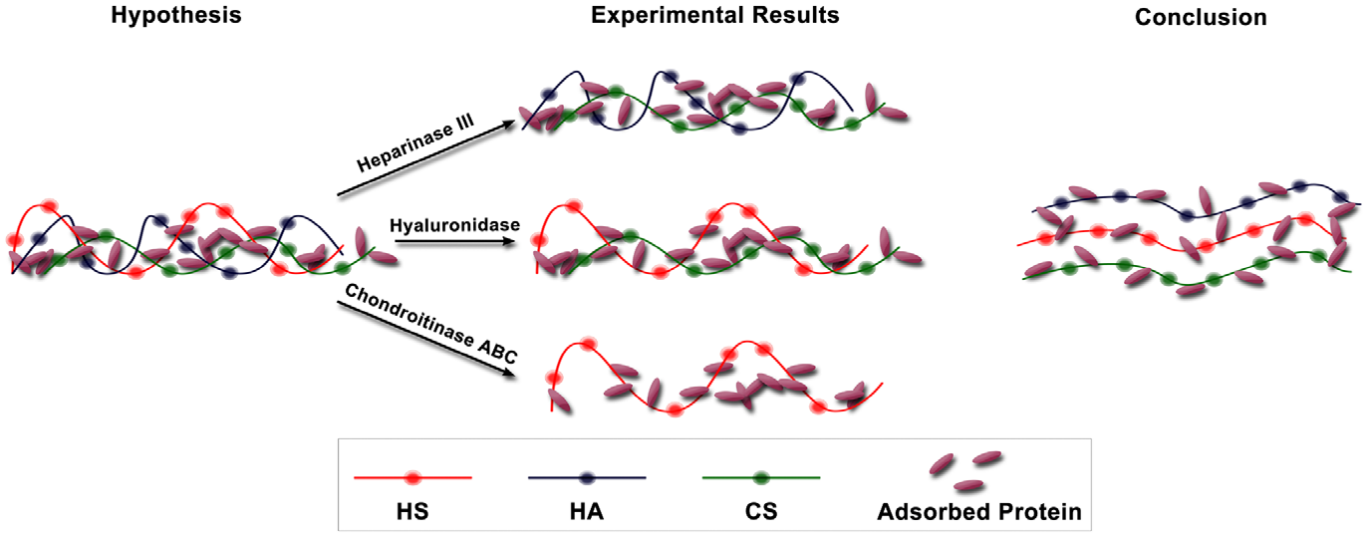
Hypothesis–Experimental results–Conclusion. Left: Our hypothesis of an interacting mesh of GAGs and albumin. Middle: A summary of the results of our experiments: removal of HS (heparinase III), HA (hyaluronidase), or both CS and HA (chondroitinase ABC) did not reduce the coverage or thickness of any of the remaining components, indicating that no individual component (HS, HA or CS) is required to stabilize the EGL. Right: The overarching conclusion of our study: GAG components do not interact but do provide binding sites for albumin.

### Image Quantification Analysis

Using ImageJ software (version 1.46; NIH, USA), the split and recombination (mergence) of color channels, and three-dimensional reconstruction of the Z-series stack were performed (Figure 1A, B and C). The orthogonal XZ-and YZ-slices were reconstructed through the image volume represented by the Z-series stack (Figure 1D and E). The maximum intensity Z-projection of the green Z-series stack (Alexa Fluor 488 channel) was created, in which each pixel contains the maximum value over all images in the stack at the particular pixel location, showing all the staining in a given FOV (Figure 1F).

#### Background noise and threshold

The pixel intensity histograms from the FOV of max-intensity Z-projection images of control and negative control were obtained using the ImageJ histogram tool, and converted to curves (Figure 1G). The maximum intensity value in 8-bit images was 255. The point of intersection between the curves was defined as the background noise value (threshold). All information equal to or higher than background was selected as a region of interest (ROI). All information lower than background was ignored during further image analysis.

#### Coverage calculation

The threshold was defined as above. The area of the ROI in the max-intensity Z-projection image was calculated using ImageJ. The percent coverage was defined as the ratio of the area of the ROI and FOV,
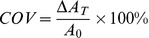
(1)where 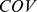 is the coverage, 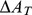 is the area of the ROI obtained at threshold, 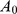 is the area of the FOV. If the ROI is not selected at threshold, the coverage is undervalued (Figure 1H).

In fact, different threshold values were obtained in different pairs of control and negative control images for the same staining, but no significant difference was found between the coverage using those different threshold values.

#### Average thickness calculation

The area and length of the bounding rectangle of the ROI in XZ-and YZ-slices were also calculated using the same threshold values as above (Figure 1I and J). The average thickness can be computed by
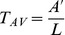
(2)where 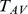 is the average thickness, and 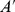 and respectively represent the area and the length of the ROI obtained at threshold.

#### Junctional thickness calculation

Combining the nuclei staining image (Figure 1C) and the max-intensity Z-projection image (Figure 1F), the outlines of cell-cell junctions are found. Then, the junction positions in XZ-and YZ-slices can be indicated (Figure 1D and E). Figure 1K shows a partial enlargement of the XZ-slice image. Junctional thickness was determined using a line in the z direction at the junction position in the XZ-and YZ-slices. The intensities of pixels along the selected lines were obtained using the ImageJ plot profile tool. As shown in Figure1L, the ordinate is the pixel intensity and the abscissa is the distance along the line. According to the SUSAN edge finding algorithm [41], the half maximum intensity value was employed as the second threshold (2^nd^ threshold) for junctional thickness detection [42], [43]. If the 2^nd^ threshold value was lower than the background threshold value, the line was considered empty so that the junctional thickness was equal to zero. However, if the 2^nd^ threshold was above the background threshold, the width of the intensity-distance curve at the 2^nd^ threshold was taken as the junction thickness. The interval between slices defined the pixel dimension in the Z direction, so that the lowest thickness that could be detected was either 0.3 or 0.36 µm.

For the correction, all of the 2^nd^ threshold values were recorded. The signal-to-noise ratio was defined as the average 2^nd^ threshold value divided by the background threshold value (noise value) [42]. Lines with a signal-to-noise ratio below 2.5 [42] overestimated junctional thicknesses and were not used for further analysis.

#### Enzyme spatial digestibility

The coverage and thickness of glycocalyx components (HS, CS, HA, and BSA) as a function of enzyme concentration (*x*) were curve fit with exponential equations: 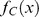 and 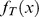. To further analyze the changes in spatial distribution of glycocalyx components after corresponding enzyme removal, we calculated the enzyme spatial digestibility (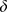). It is defined as:
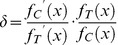
(3)where 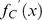 and 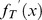 are the first derivatives of 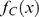 and 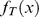 that denote the coverage (%) and thickness (µm) of the glycocalyx component, respectively. 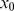 is the starting enzyme concentration.

### Statistical Analysis

Data are presented as means ± SD obtained from at least three independent experiments. The images for coverage and average thickness calculations were obtained from respectively, at least 18 max-intensity Z-projection images and 180 X-and Y-slices. For junctional thickness calculations, lines were selected for at least 180 junction positions from the X-and Y-slices. Statistical analysis was performed by one-way analysis of variance (ANOVA) with either the least significant difference (LSD) test or Tamhane’s T^2^ test (depending on Levene’s statistic for homogeneity of variance) using the SPSS 20.0 software package. Differences in means were considered significant if *P*<0.05. All images shown in the present paper had the background subtracted unless indicated otherwise.

## Results and Analysis

### Enzymatic Removal of HS by Heparinase III

The dose-dependent removal of HS by heparinase III is shown in Figure 2. The initial coverage of HS on RFPECs (83.3±3.2%, control) was significantly decreased to 72.1±3.5, 65.0±3.8, 42.0±4.3, 14.5±5.1, 5.8±0.9, and 2.6±1.0% in the presence of 15, 45, 135, 405, 1215 and 3645 mU/mL heparinase III, respectively. The average thickness and junctional thickness of HS (2.2±0.6 and 3.2±0.4 µm, respectively) were significantly decreased when the heparinase III concentrations were above the 45 mU/mL. At the highest enzyme concentration, the average thickness of HS decreased by 54.9%, and the junctional thickness of HS decreased by 55.0%. There were no significant changes in coverage, average thickness and junctional thickness between 1215 and 3645 mU/mL, indicating that 1215 mU/mL heparinase III could remove most of the HS.

The equations describing the coverage and average thickness of HS against the concentration of heparinase III (*x*), were given by
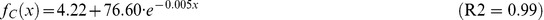
(4)

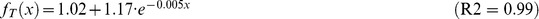
(5)The enzyme spatial digestibility (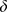) was 1.73, independent of enzyme concentration, indicating that the reduction of HS in coverage was 1.73 times greater than in average thickness.

### HA after Heparinase III Treatment

The coverage, average thickness, and junctional thickness of HA were 88.8±6.4%, 1.9±0.3 and 2.1±0.3 µm, respectively after treatment with 1215 mU/mL heparinase III (Figure 3A–C; Figure S1A shows immuno-staining images). There were no statistically significant changes in the coverage, average thickness, and junctional thickness of HA after heparinase III treatment at this dose that removed virtually all of the HS, suggesting little interaction between HA and HS.

### CS Clusters Near the Junctions after Heparinase III Treatment

The structure of CS after heparinase III treatment is shown in Figure 3D–F (Figure S1B shows immuno-staining images). Before enzyme treatment, the coverage, average thickness and junctional thickness of CS were 87.2±5.3%, 1.2±0.2 and 1.7±0.3 µm, respectively. After heparinase III treatment at a high dose, the coverage of CS had no significant change, but the average thickness and junctional thickness of CS actually increased significantly by 7.3% and 26.1%, respectively, in the presence of 1215 mU/mL heparinase III. These results indicate that CS clusters near the junctions after removal of HS. There was no evidence of CS collapse as HS was depleted.

### Adsorbed Albumin after Heparinase III Treatment

The coverage, average thickness, and junctional thickness of albumin before enzyme treatment were 93.1±5.4%, 1.8±0.5 and 2.6±0.6 µm, respectively (Figure 3G–I; Figure S1C shows immuno-staining images). There were no significant changes in coverage, average thickness and junctional thickness after heparinase III treatment at both 135 and 1215 mU/mL.

### Enzymatic Removal of HA by Hyaluronidase

The dose-dependent removal of HA by hyaluronidase is shown in Figure 4. Before enzyme treatment, the coverage, average thickness and junctional thickness of HA were 87.7±3.3%, 1.9±0.3 and 2.1±0.2 µm, respectively. The coverage, average thickness, and junctional thickness of HA were significantly decreased by 41.6, 26.9, and 25.0%, respectively in the presence of 1.5 U/mL hyaluronidase, and by 93.1, 56.0 and 53.3%, respectively in the presence of 4.5 U/mL hyaluronidase. There were no significant changes in coverage, average thickness, and junctional thickness between 4.5 and 13.5 U/mL hyaluronidase, suggesting that 4.5 mU/mL could remove most of the HA.

The equations describing the coverage and average thickness of HA as a function of hyaluronidase concentration (*x*), were given by
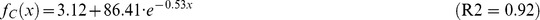
(6)

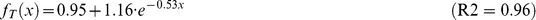
(7)


The enzyme spatial digestibility (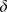) was 1.60, independent of enzyme concentration, indicating that the decrease of HA in coverage was 1.60 times greater than in average thickness.

### The Structure of HS, CS and Adsorbed Albumin after Hyaluronidase Treatment

No significant differences were found between the hyaluronidase-treated and control groups for the coverage, average thickness, and junctional thickness of HS, CS and albumin (Figure 5, Figure S2 shows immuno-staining images).

### Enzymatic Removal of CS by Chondroitinase ABC

The removal of CS by chondroitinase ABC was also dose-dependent (Figure 6). The initial coverage of CS on RFPECs (86.5±5.6%) was significantly decreased to 61.2±6.5, 36.8±4.6, 19.1±6.3, and 16.5±5.4% in the presence of 15, 45, 135, and 405 mU/mL chondroitinase ABC, respectively. The average thickness (1.2±0.2 µm) was significantly decreased by 8.7, 19.0, 26.7, and 31.3%, respectively, in the presence of 15, 45, 135, and 405 mU/mL chondroitinase ABC. The junctional thickness (1.8±0.3 µm) was significantly decreased by 13.7, 26.0, 40.8, and 42.8%, respectively, in the presence of 15, 45, 135, and 405 mU/mL chondroitinase ABC. There were no significant changes in the coverage, average thickness, and junctional thickness between 135 and 405 mU/mL chondroitinase ABC, suggesting that the 135 mU/mL could remove most of the CS.

The equations describing the coverage and average thickness of CS as a function of chondroitinase ABC concentration (*x*), were given by
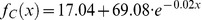
(8)

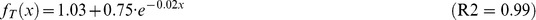
(9)The enzyme spatial digestibility (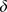) was 1.28, independent of enzyme concentration, indicating that the reduction of CS in coverage was 1.28 times greater than in average thickness.

### Degradation of HA by Chondroitinase ABC

The HA was also degraded by chondroitinase ABC as shown in Figure 7A–C (Figure S3A shows immuno-staining images). The coverage, average thickness, and junctional thickness of HA were decreased by 90.9, 54.8, and 53.2%, respectively, in the presence of 15 mU/mL chondroitinase ABC. In the presence of 45 mU/mL chondroitinase ABC induced no further changes. Because a low dose of chondroitinase ABC almost completely cleaved HA, the enzyme spatial digestibility of HA was not calculated. But it is clear that the coverage was reduced more extensively than the average thickness.

### The Structure of HS after Chondroitinase ABC Treatment

To investigate whether chondroitinase ABC would affect the glycocalyx structure, the visualization of HS was performed in the presence of 0, 15 and 405 mU/mL chondroitinase ABC (Figure 7D–F; Figure S3B shows immuno-staining images). The coverage, average thickness, and junctional thickness of HS were not significantly changed, suggesting that direct removal of CS does not affect the HS.

### The Structure of Adsorbed Albumin after Chondroitinase ABC Treatment

Figure 7G–I (Figure S3C displays immuno-staining images) shows the changes of adsorbed albumin after chondroitinase ABC treatment. The coverage of albumin was not changed significantly. In the presence of 15 and 405 mU/mL chondroitinase ABC, the changes in the average and junctional thickness were statistically significant, although the changes were relatively small (15% reduction of the average thickness and 20.8% reduction of the junctional thickness at 405 mU/mL).

## Discussion

We have, in recent years, analyzed the function and organization of the EGL on various cells and animal tissues [16], [31], [44], [45]. Modifications of EC function after enzyme treatment of the EGL, include mechanotransduction [3], [25], permeability [2], [11] and adhesion [46]. But, the structural changes of the EGL after enzyme treatment that may underlie the functional modifications have not been thoroughly investigated as summarized in Table 1. A review of this table demonstrates that reductions of fluorescence intensity and thickness of the EGL have been observed at various doses of enzyme using several different visualization methods. But the effects of specific enzymes on off-target components have not been determined, leaving the structure of the EGL unclear. The present study demonstrates the organization and spatial distribution characteristics of the EGL after removal of prominent GAGs. The interaction of GAGs and their contributions to structural stability of the EGL are revealed. The study was based on RFPECs because these ECs displayed the most immune-positivity over the complete series of GAG antibodies employed.

The control experiments labeling the glycocalyx components on RFPECs using confocal laser scanning microscopy with immunofluorescence staining in the absence of enzyme showed that the coverage of HS, CS, HA, and adsorbed albumin was 83.3±3.2, 87.2±5.3, 87.6±3.3 and 93.1±5.4%, respectively, indicating similar high levels of coverage of each GAG. But we did not do any co-staining for more than one GAG or albumin. It is very likely that the area not covered by a particular GAG/albumin (7% to 17%) is covered by one or more of the other GAGs/albumin. Co-staining studies were not performed as the best secondary antibodies were all based on Alexa Fluor 488. The same approach used on the common carotid artery of C57BL/6J (B6) mice showed a similar coverage of HS and HA, which respectively were 86±9 and 85±6% [47]. Our results show that the average thickness of HS, CS, HA, and adsorbed albumin on RFPECs was 2.2±0.6, 1.2±0.2, 1.9±0.3 and 1.8±0.5 µm, and the junctional thickness was 3.2±0.4, 1.7±0.3, 2.1±0.3 and 2.6±0.6 µm, respectively. A nonuniform thickness of individual GAGs on RFPECs was found, which is similar to others [42], [43], [48]. Although the detailed ultrastructure of the EGL and its components is difficult to discern from these immunofluorescence staining-confocal microscopy studies, since the coverages and thicknesses of all components were similar, we can hypothesize that the GAG components and adsorbed albumin were well integrated within the structure of the EGL.

Heparinase III, hyaluronidase, and chondroitinase ABC, were used to remove respective GAGs by varying the incubation concentration for 2 hr. Heparinase III cleaves HS exclusively, while hyaluronidase from *Streptomyces hyalurolyticus* is specific for HA. The chondroitinase ABC (EC 4.2.2.4) degrades both CS and HA. It has been reported that HA degradation by chondroitinase ABC is most effective at a pH near 6.8 [39], and less efficient at pH 8.6 [49], [50] or 9.1 [51], [52], while according to the instructions from the manufacturer (Sigma), chondroitinase ABC degrades HA and CS optimally at pH 6.2 and 8.0. To be consistent with the cell culture conditions, all enzymes were prepared at pH 7.3. Our results demonstrate that the effect of heparinase III, hyaluronidase, and chondroitinase ABC on their target GAGs is dose-dependent. 1215 mU/mL heparinase III removes most of the HS (Figure 2); 4.5 U/mL hyaluronidase and 15 mU/mL chondroitinase ABC remove most of the HA (Figures 4 and 6); and 405 mU/mL chondroitinase ABC removes most of the CS (Figure 6). The observation that chondroitinase removes both CS and HA has been reported in other studies [39], [52]. The degradation of HA by chondroitinase occurs at a substantially lower concentration of enzyme than the degradation of CS.

To further analyze the changes in spatial distribution of GAGs after corresponding enzyme treatment, we calculated the enzyme spatial digestibility. We found that the enzyme spatial digestibility of HS, CS, and HA were 1.73, 1.60, and 1.28, respectively, indicating that the remaining fractions of target component did not collapse after reduction in coverage.

After nearly complete removal HS by 135 and 1215 mU/mL heparinase III, the HA and adsorbed albumin were not significantly different from the control state in the absence of enzyme (Figures 3A–C and G–I). The average and junctional thickness of CS actually significantly increased by 7.3 and 26.1%, respectively, in the presence of 1215 mU/mL heparinase III (Figure 3E and F). These data show that CS clusters near the junctions after removal of HS and that removal of HS does not lead to the removal or collapse of any other component, suggesting that the stability of the EGL is independent of HS.

After almost complete removal of HA by 1.5 and 4.5 U/mL hyaluronidase, HS, CS, and adsorbed albumin were not affected in their coverage and average or junctional thickness (Figure 5). These data indicate that removal of HA does not lead to the removal or collapse of any other component, indicating that EGL stability is independent of HA. However, 15 and 405 mU/mL chondroitinase ABC that degraded most of HA and CS caused the thickness of adsorbed albumin to be decreased by a relatively small but statistically significant amount (Figure 7H and I). Taken together, these results indicate that the decrease in thickness of adsorbed albumin by chondroitinase is due to the degradation of both CS and HA, removing many binding sites for the capture of albumin. Surprisingly, neither hyaluronidase nor chondroitinase ABC affected the organization of HS (Figures 5A–C and 7D–F).

In summary, the structural changes of the EGL after various enzyme treatments have been studied systematically to improve our understanding of the organization of this gel-like mesh. The following conclusions have been extracted from the data:

1. The high level of coverage of individual GAGs and adsorbed albumin in the absence of enzyme indicate that all GAGs and adsorbed albumin are well inter-mixed within the structure of the EGL.
2. Structural changes of individual GAGs are dependent upon the dose of their specific enzyme, and decrease much more in coverage than in thickness after enzyme treatment.
3. The removal of a single GAG component by a specific enzyme does not degrade or collapse other GAGs, suggesting a structural stability of the EGL that is independent of the interaction between individual GAG components. A summary of our results in this regard is presented in schematic form in Figure 8. There we show our hypothesis of an interacting mesh of GAGs and albumin at the left. The results of our experiments are summarized in the middle showing that removal of either HS or HA did not reduce the coverage or thickness of any of the remaining components, indicating that neither HS nor HA is required to stabilize the EGL. Although removal of CS and HA with chondroitinase did not reduce the coverage or thickness of HS, it did reduce adsorbed albumin, although the effect was not large. The overarching conclusion of our study is depicted at the right of Figure 8 that suggests non-interacting GAG components that each provides binding sites for albumin. The influence of albumin concentration in the media on the stability of GAG components has not been investigated in the present study.

## Supporting Information

Figure S1 **The immunofluorescence staining images of CS, HA, and adsorbed albumin on heparinase III-treated RFPECs.** (A) CS; (B) HA; (C) adsorbed albumin. In each group, Top: Z-projection; bottom: cross-sectional images of stack along the dashed line. The arrow head indicates the cell-cell junction. Scale bar: 20 µm.
(TIF)

Figure S2 **The immunofluorescence staining images of HS, CS, and adsorbed albumin on hyaluronidase-treated RFPECs.** (A) HS; (B) CS; (C) adsorbed albumin. In each group, Top: Z-projection; bottom: cross-sectional images of stack along the dashed line. The arrow head indicates the cell-cell junction. Scale bar: 20 µm.
(TIF)

Figure S3 **The immunofluorescence staining images of HA, HS, and adsorbed albumin on chondroitinase ABC-treated RFPECs.** (A) HA; (B) HS; (C) adsorbed albumin. In each group, Top: Z-projection; bottom: cross-sectional images of stack along the dashed line. The arrow head indicates the cell-cell junction. Scale bar: 20 µm.
(TIF)

Figure S4 **Effects of heparinase III on 10E4 epitope and Hepss-1 epitope anti-HS antibody–labeled HS.** Both 10E4 epitope and HepSS-1 epitope anti-HS antibody**–**labeled HS were also almost completely removed by 1215 mU/mL heparinase III (2 hr). **P<0.01.
(TIF)

Figure S5 **The immunofluorescence staining images of CS using the CS-56 antibody.** The strong CS-56 immunopositivity remained on the cell surface after 1215 mU/mL chondroitinase ABC digestion. A representative experiment is shown (background was removed). Scale bar: 20 µm.
(TIF)

Figure S6 **The immunofluorescence staining images of HA using the HABP from US biological.** Using the HABP from US biological, HA staining was actually enhanced by hyaluronidase treatment at 4.5 and 13.5 U/ml. A representative experiment is shown. Scale bar: 20 µm.
(TIF)

Discussion S1 **Supplementary Discussion.**
(DOC)
