# Supplementary figures and images for: The Structural Stability of the Endothelial Glycocalyx after Enzymatic Removal of Glycosaminoglycans
Source: PLoS One. 2012 Aug 14;7(8):e43168. doi: 10.1371/journal.pone.0043168 (PMC3419189; doi:10.1371/journal.pone.0043168)

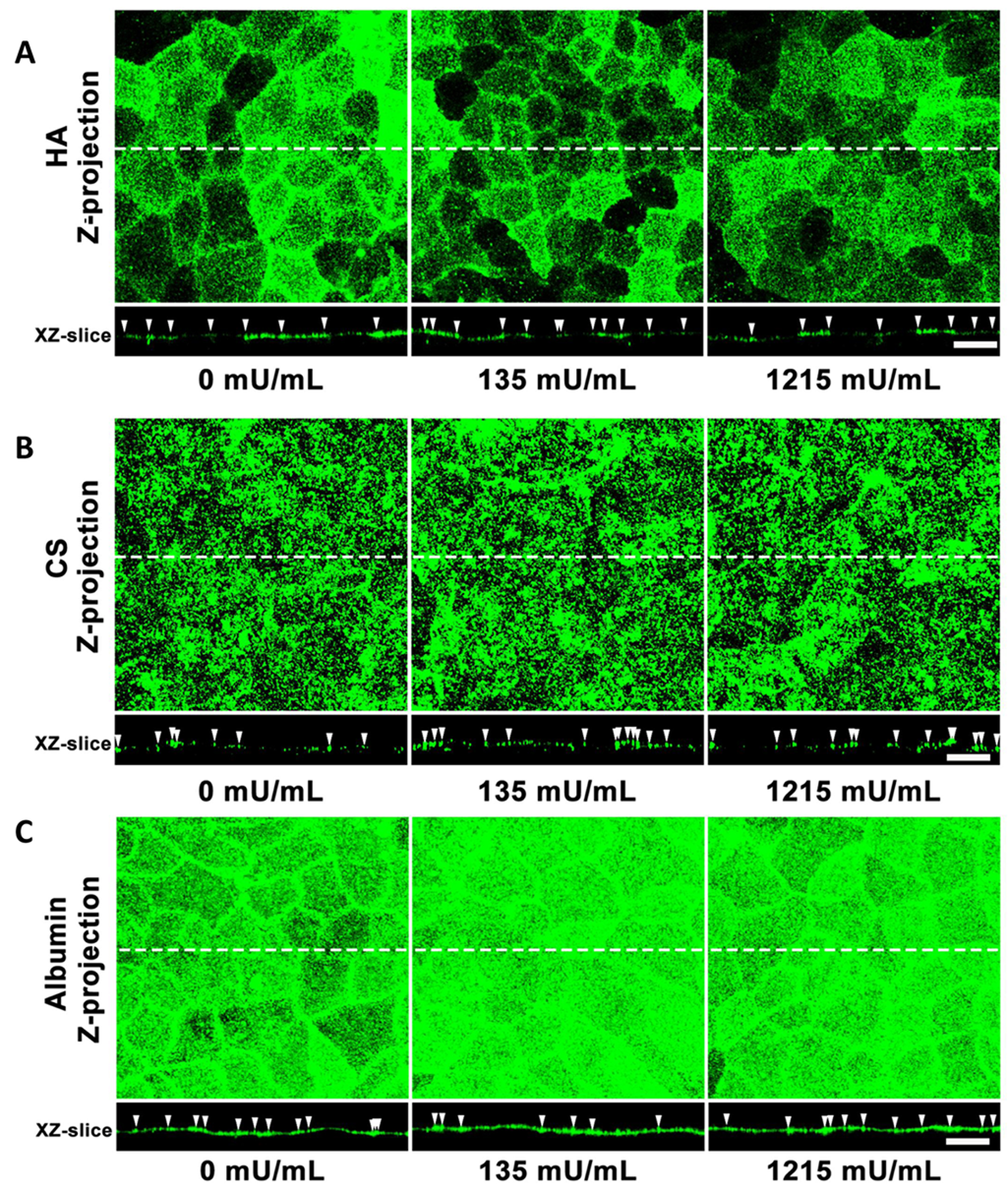

Supplement: Figure S1 — The immunofluorescence staining images of CS, HA, and adsorbed albumin on heparinase III-treated RFPECs. (A) CS; (B) HA; (C) adsorbed albumin. In each group, Top: Z-projection; bottom: cross-sectional images of stack along the dashed line. The arrow head indicates the cell-cell junction. Scale bar: 20 µm. (TIF) [file pone.0043168.s001.tif]

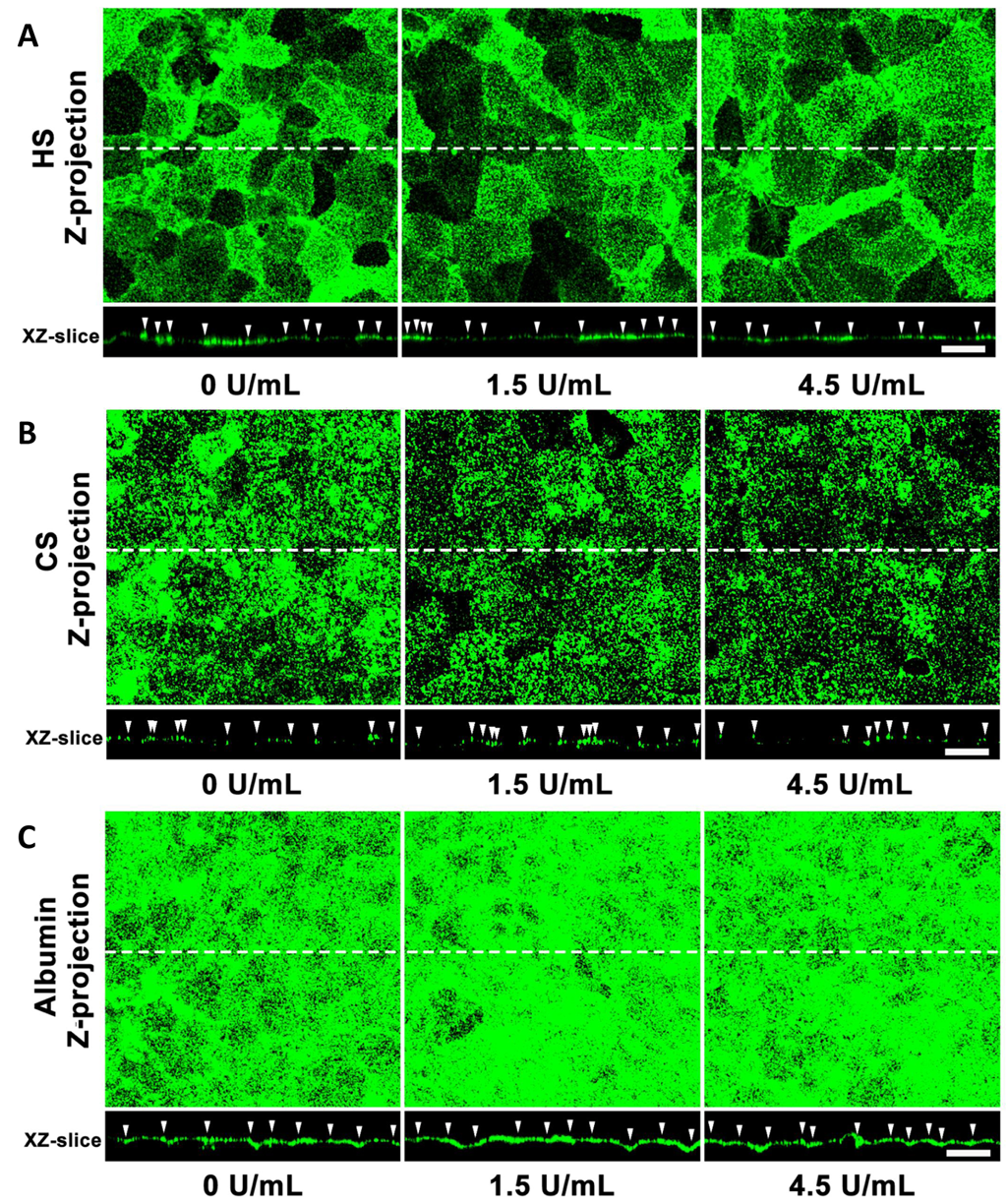

Supplement: Figure S2 — The immunofluorescence staining images of HS, CS, and adsorbed albumin on hyaluronidase-treated RFPECs. (A) HS; (B) CS; (C) adsorbed albumin. In each group, Top: Z-projection; bottom: cross-sectional images of stack along the dashed line. The arrow head indicates the cell-cell junction. Scale bar: 20 µm. (TIF) [file pone.0043168.s002.tif]

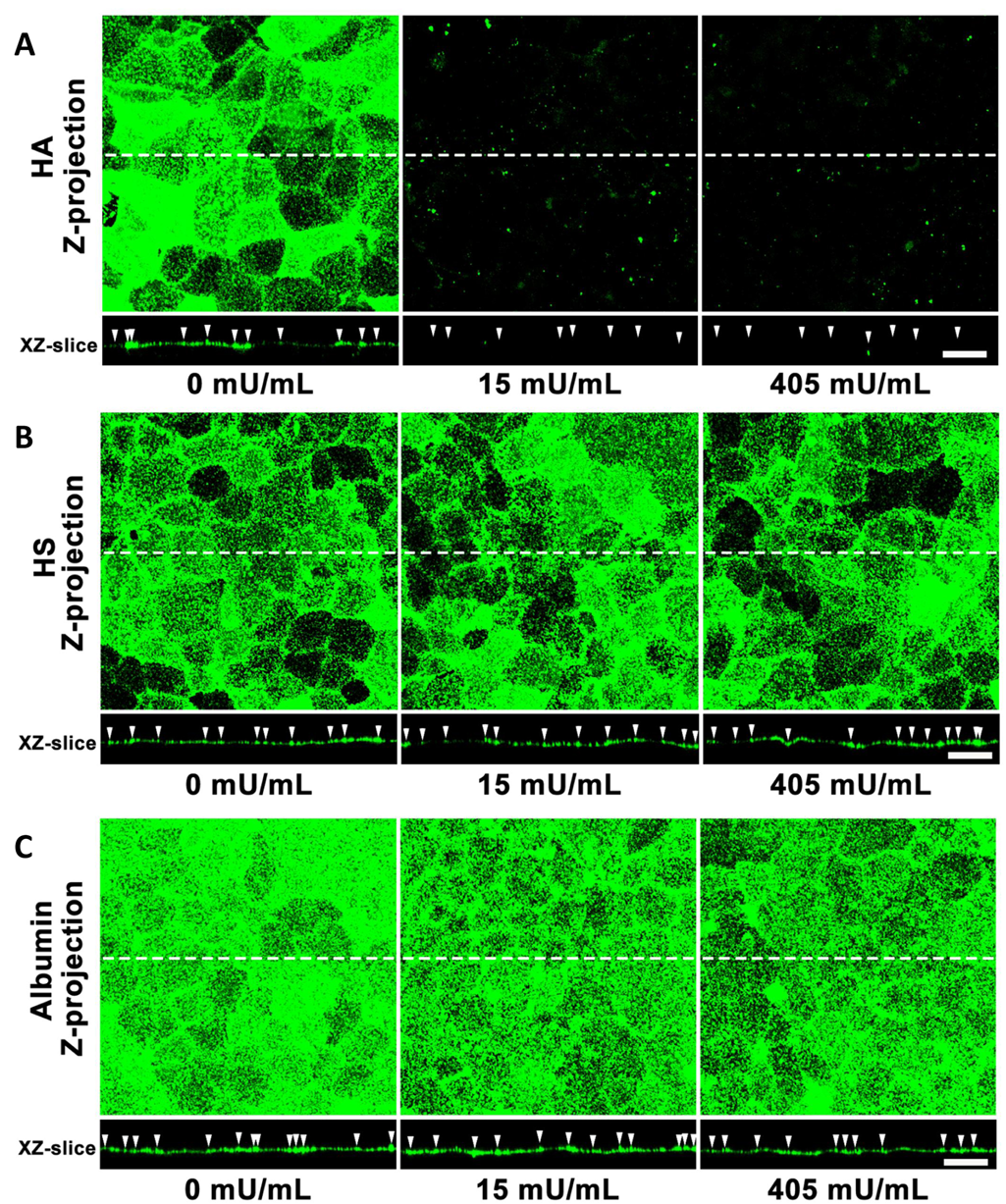

Supplement: Figure S3 — The immunofluorescence staining images of HA, HS, and adsorbed albumin on chondroitinase ABC-treated RFPECs. (A) HA; (B) HS; (C) adsorbed albumin. In each group, Top: Z-projection; bottom: cross-sectional images of stack along the dashed line. The arrow head indicates the cell-cell junction. Scale bar: 20 µm. (TIF) [file pone.0043168.s003.tif]

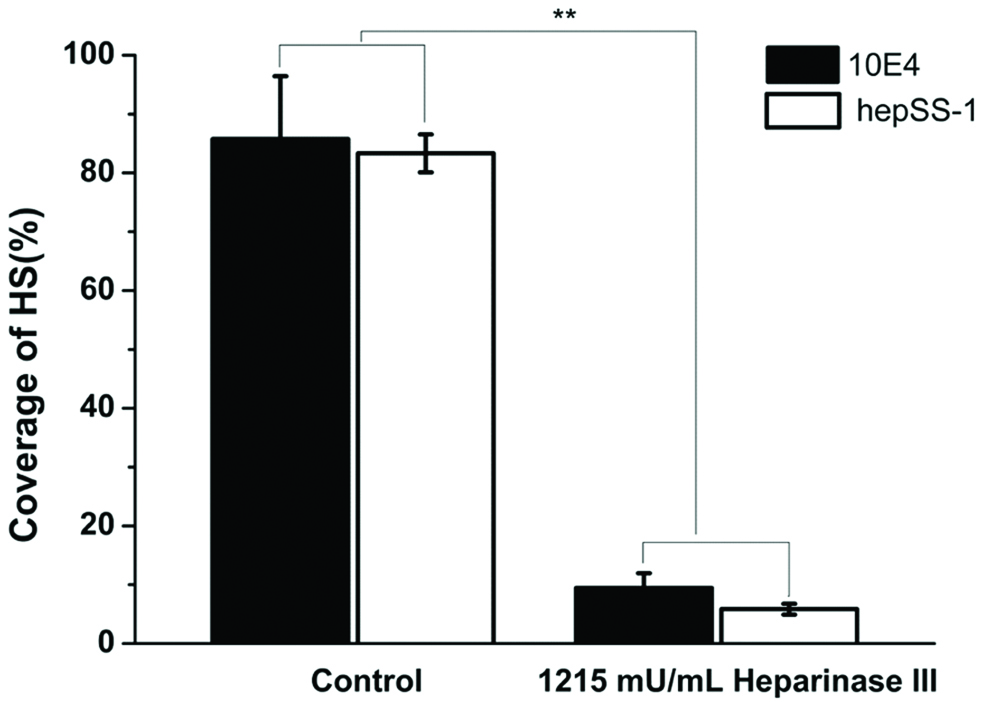

Supplement: Figure S4 — Effects of heparinase III on 10E4 epitope and Hepss-1 epitope anti-HS antibody–labeled HS. Both 10E4 epitope and HepSS-1 epitope anti-HS antibody–labeled HS were also almost completely removed by 1215 mU/mL heparinase III (2 hr). **P<0.01. (TIF) [file pone.0043168.s004.tif]

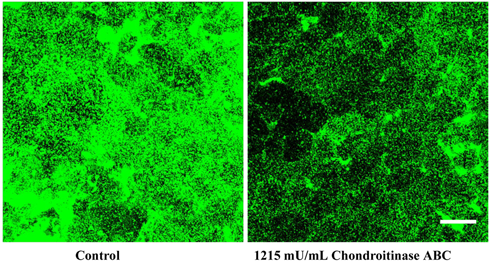

Supplement: Figure S5 — The immunofluorescence staining images of CS using the CS-56 antibody. The strong CS-56 immunopositivity remained on the cell surface after 1215 mU/mL chondroitinase ABC digestion. A representative experiment is shown (background was removed). Scale bar: 20 µm. (TIF) [file pone.0043168.s005.tif]

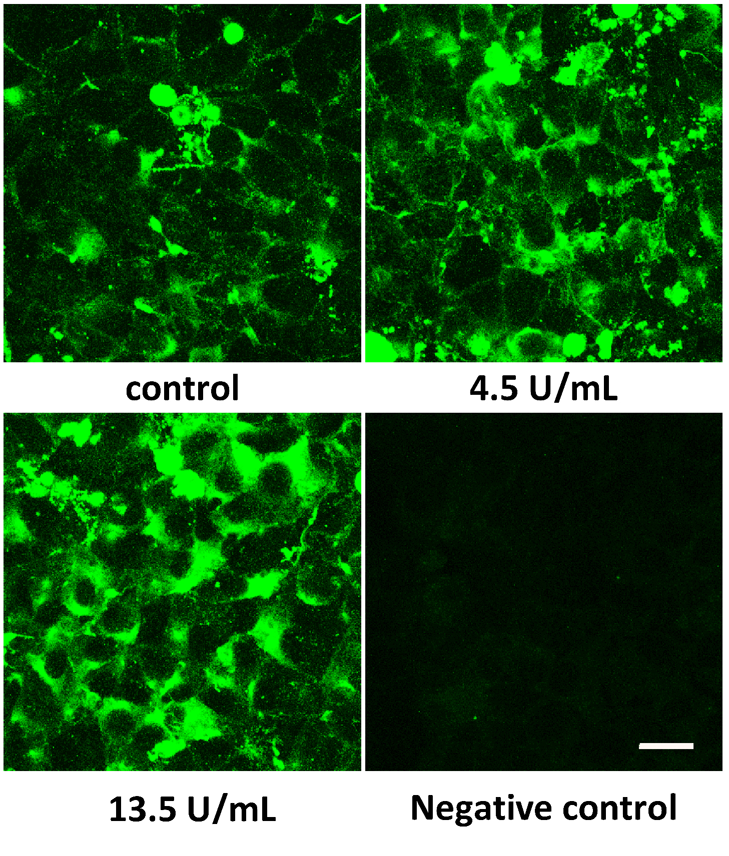

Supplement: Figure S6 — The immunofluorescence staining images of HA using the HABP from US biological. Using the HABP from US biological, HA staining was actually enhanced by hyaluronidase treatment at 4.5 and 13.5 U/ml. A representative experiment is shown. Scale bar: 20 µm. (TIF) [file pone.0043168.s006.tif]
