# Supplementary material for: The Structural Stability of the Endothelial Glycocalyx after Enzymatic Removal of Glycosaminoglycans
Source: PLoS One. 2012 Aug 14;7(8):e43168. doi: 10.1371/journal.pone.0043168 (PMC3419189; doi:10.1371/journal.pone.0043168)
Supplement: Discussion S1 — Supplementary Discussion. (DOC) [file pone.0043168.s007.doc]

**Supplementary Discussion**

Figures S1-S3 display representative immuno-staining images of HS, CS, HA, and albumin that correspond to the conditions described in Figs. 3, 5, and 7 in the paper.

It has been suggested that the epitope profile, obtained with different antibodies, can be used as an indirect measure of the sequence variability in GAGs [1,2]. We presently used both 10E4 (Figs. S2A, S3B) and hepSS-1 (Fig. 2) epitope anti-HS antibodies to label the HS on RFPECs, and obtained no significant difference between the structures. The labeled HS using either antibody was almost completely removed by 1215 mU/mL heparinase III (see Fig. S4).

For the labeling of CS, we initially used a mouse monoclonal anti-CS antibody (clone CS-56, Sigma) and obtained good staining, but unfortunately, the strong staining remained on the cell surface after chondroitinase ABC treatment, giving the false impression that chondroitinase ABC did not fully degrade CS (See Fig. S5). Recently, it was also observed that in the chondroitionase ABC infused rat hindbrain ventricular surface, the strong CS-56 immunopositivity remained [3]. However, when the *B.simplicifolia* lectin II (GSL II) was used for the staining, we found CS was completely removed by chondroitinase ABC. It is unlikely that CS is protected from enzymatic digestion via interaction with other molecules [3]. Rather, it is more likely that CS is degraded by the enzyme but the fragments are retained on the cell surface [1]. The HABP has been successfully used for detection of HA in cell cultures as well as in tissue sections [4]. We presently used it to label HA on RFPECs. According to the instructions from the manufacturer (EMD Chemicals), the HABP is composed of two binding polypeptides, which are derived from N-terminal regions of the HA binding proteoglycan (PG) and link proteins. Link proteins stabilize the binding of PG monomers to HA by binding simultaneously to HA and to the HA-binding region of PG monomer core proteins [5]. In particular, the binding of HABP to HA on the cell surface could be inhibited by soluble HA and by the HA decasaccharide (the minimum size of an HA oligosaccharide) [6,7]. In addition, partial or complete saturation of HA by HA binding molecules such as CD44 [8] and versican [9], blocks staining [10]. We found that HA was only labeled in some areas of the RFPECs surface. The results shown in the present paper overestimate the overall coverage of HA, because only areas with positive staining were considered (less than 30% of the entire cell surface in the absence of enzyme, data not shown). Unlike the HABP from EMD Chemicals, the one from US biological (not used) labeled the intracellular HA, showing strong staining even after degradation by hyaluronidase (See Fig. S6), suggesting that internalization of HA accompanied its degradation. It is known that ECs are capable of secreting, binding, endocytosing and degrading HA [8,11-13].

**Reference**

1. Rosen M, Edfors-Lilja I, Bjornsson S (2002) Quantitation of repetitive epitopes in glycosaminoglycans immobilized on hydrophobic membranes treated with cationic detergents. Anal Biochem 308: 210-222.

2. Bjornsson S (2000) Detection of epitopes in glycosaminoglycans immobilized on hydrophobic membranes. Scand J Clin Lab Invest Suppl 233: 27-35.

3. Kwok JC, Yuen YL, Lau WK, Zhang FX, Fawcett JW, et al. (2012) Chondroitin sulfates in the developing rat hindbrain confine commissural projections of vestibular nuclear neurons. Neural Dev 7: 6.

4. de la Motte CA, Drazba JA (2011) Viewing hyaluronan: imaging contributes to imagining new roles for this amazing matrix polymer. J Histochem Cytochem 59: 252-257.

5. Tang LH, Rosenberg L, Reiner A, Poole AR (1979) Proteoglycans from bovine nasal cartilage. Properties of a soluble form of link protein. J Biol Chem 254: 10523-10531.

6. Tengblad A (1981) A comparative study of the binding of cartilage link protein and the hyaluronate-binding region of the cartilage proteoglycan to hyaluronate-substituted Sepharose gel. Biochem J 199: 297-305.

7. Rosenberg L, Tang LH, Pal S, Johnson TL, Choi HU (1988) Proteoglycans of bovine articular cartilage. Studies of the direct interaction of link protein with hyaluronate in the absence of proteoglycan monomer. J Biol Chem 263: 18071-18077.

8. Gaffney J, Matou-Nasri S, Grau-Olivares M, Slevin M (2010) Therapeutic applications of hyaluronan. Mol Biosyst 6: 437-443.

9. Theocharis AD, Skandalis SS, Tzanakakis GN, Karamanos NK (2010) Proteoglycans in health and disease: novel roles for proteoglycans in malignancy and their pharmacological targeting. FEBS J 277: 3904-3923.

10. Eggli PS, Graber W (1995) Association of hyaluronan with rat vascular endothelial and smooth muscle cells. J Histochem Cytochem 43: 689-697.

11. Wheeler-Jones CP, Farrar CE, Pitsillides AA (2010) Targeting hyaluronan of the endothelial glycocalyx for therapeutic intervention. Curr Opin Investig Drugs 11: 997-1006.

12. Lennon FE, Singleton PA (2011) Hyaluronan regulation of vascular integrity. Am J Cardiovasc Dis 1: 200-213.

13. Laurent TC, Fraser JR (1992) Hyaluronan. FASEB J 6: 2397-2404.
